# Cross-Talk between Oxysterols and Glucocorticoids: Differential Regulation of Secreted Phopholipase A2 and Impact on Oligodendrocyte Death

**DOI:** 10.1371/journal.pone.0008080

**Published:** 2009-11-26

**Authors:** Amalia Trousson, Joelle Makoukji, Patrice X. Petit, Sophie Bernard, Christian Slomianny, Michael Schumacher, Charbel Massaad

**Affiliations:** 1 UMR788, Inserm and University Paris-Sud 11, IFR 93, Le Kremlin-Bicêtre, France; 2 UPR 2228, CNRS and University Paris Descartes, IFR95, Paris, France; 3 Cancer, Apoptosis, and Mitochondria Team, UMR8104 CNRS, Institut Cochin, Paris, France; 4 Inserm U800 and University Lille 1, Villeneuve d'Ascq, France; University of North Dakota, United States of America

## Abstract

**Background:**

Oxysterols are oxidized forms of cholesterol. They have been shown to be implicated in cholesterol turnover, inflammation and in neurodegenerative diseases such as Alzheimer's disease and multiple sclerosis. Glial cells are targets of oxysterols: they inhibit astrocyte proliferation after brain injury, and we have previously shown that 25-hydroxycholesterol (25OH) provokes oligodendrocyte apoptosis and stimulates the expression of sPLA2 type IIA (sPLA2-IIA), which has a protective effect.

**Methodology/Principal Findings:**

As glucocorticoids are well-known for their anti-inflammatory effects, our aim was to understand their direct effects on oxysterol-induced responses in oligodendrocytes (sPLA2-IIA stimulation and apoptosis). We demonstrate that the synthetic glucocorticoid dexamethasone (Dex) abolishes the stimulation of sPLA2-IIA by 25-hydroxycholesterol (25-OH). This inhibition is mediated by the glucocorticoid receptor (GR), which decreases the expression of the oxysterol receptor Pregnane X Receptor (PXR) and interferes with oxysterol signaling by recruiting a common limiting coactivator PGC1α. Consistent with the finding that sPLA2-IIA can partially protect oligodendrocytes against oxysterol-triggered apoptosis, we demonstrate here that the inhibition of sPLA2-IIA by Dex accelerates the apoptotic phenomenon, leading to a shift towards necrosis. We have shown by atomic force microscopy and electron microscopy that 25-OH and Dex alters oligodendrocyte shape and disorganizes the cytoplasm.

**Conclusions/Significance:**

Our results provide a new understanding of the cross-talk between oxysterol and glucocorticoid signaling pathways and their respective roles in apoptosis and oligodendrocyte functions.

## Introduction

Cholesterol is predominantly located in the brain, especially in the myelin sheaths formed by oligodendrocytes [1], which are the myelinating glial cells of the central nervous system. It also exerts pleiotropic effects on brain functioning and homeostasis and gives rise to several neuroactive compounds, mainly side-chain cleavage-derived steroid hormones and oxysterols. The latter are natural compounds originating from the enzymatic oxidation of cholesterol. There are several different oxysterols, in particular 24(S)-hydroxycholesterol (24(S)-OH), 25-hydroxycholesterol (25-OH) and 27-hydroxycholesterol (27-OH), which are respectively synthesized by means of the cholesterol hydroxylase enzymes CYP46A1, 25-hydroxylase and 27-hydroxylase [2]. Oxysterols have been shown to be implicated in cholesterol turnover, inflammation and in neurodegenerative diseases such as Alzheimer's disease (AD) [3], [4] and multiple sclerosis (MS) [5], [6]. Not only neurons, but also glial cells are targets for the actions of oxysterols: they inhibit astrocyte proliferation after brain injury [7] and, as we have previously shown, can cause oligodendrocyte apoptosis [8].

Oxysterols (particularly 22(R)-OH, 24(S)-OH and 25-OH) bind and activate the two isoforms of the nuclear receptor Liver X Receptor (LXRα and LXRβ) [9] and the Pregnane X Receptor (PXR). However, not all oxysterols share the same mechanisms of action; for example, 22(S)-OH does not activate LXR [10]. While LXRβ is ubiquitously expressed, LXRα is prominently expressed in liver, kidney, intestine, adipose tissue, lungs and cerebellum. Pregnane X Receptor (PXR), a xenobiotic-activated member of the nuclear receptor superfamily, is expressed in brain, liver, kidney and lung [11], [12]. Both LXR and PXR receptors form heterodimers with retinoic X receptor (RXR), the nuclear receptor for 9-*cis* retinoic acid, and they regulate gene expression in the nucleus by interacting with specific responsive elements. After oxysterol binding, LXR-RXR or PXR-RXR dimers detach from corepressors, interact with coactivators such as p160s [13] or PGC-1α (peroxysome proliferator activator receptor γ coactivator-1α) [14] and activate target gene expression.

Glucocorticoids are very well known for their anti-inflammatory effects and are used in the treatment of inflammatory neurodegenerative diseases such as MS. Their anti-inflammatory effects are mostly due to the ability of the glucocorticoid receptor (GR) to repress pro-inflammatory gene expression by inhibiting inflammatory transcription factors such as NF-κB (Nuclear Factor κ B) or AP-1 (Activator Protein 1). Besides its direct interactions with these nuclear transcription factors, the hormone-bound GR can bind as a homodimer to glucocorticoid response elements (GRE) that are present in the vicinity of glucocorticoid-modulated gene promoters. Like the LXRs, the GR enhances transcription activity by recruiting transcription coactivators such as p160s or PGC-1α.

We have previously shown that the secreted type IIA phospholipase A2 (sPLA2-IIA) is stimulated by oxysterols in the oligodendrocyte cell line 158N, suggesting a tight involvement of myelinating glial cells in inflammatory processes [8]. Indeed, sPLA2 is implicated in various inflammatory diseases such as atherosclerosis and endotoxic shock [15]. In the CNS, sPLA2-IIA is involved in inflammation [16] and can induce neuronal cell death [17]. In AD, for example, sPLA2-IIA is up-regulated [18]. However, sPLA2 activity is not restricted to apoptosis, as it also exerts beneficial actions: it has proliferative effects towards smooth muscle cells [19], [20,], uterine cells [21], monocytes [22] and it can also exert neurotrophic effects, as has been demonstrated for cerebellar granule neurons [23]. In oligodendrocytes, we have demonstrated a protective role of sPLA2-IIA against oxysterol-triggered apoptosis [8].

Remarkably, as we have previously demonstrated, oxysterols can exert dual actions in oligodendrocytes [8]. On the one hand, they can indeed induce the apoptosis of oligodendrocytes, but on the other hand, they also stimulate the inflammatory sPLA2-IIA, which partially protects oligodendrocytes from 25-OH-triggered cell death. The aim of this work was to examine the influence of glucocorticoids on oxysterol effects in oligodendrocytes. We demonstrate that the stimulation of sPLA2-IIA by oxysterols can be blocked by glucocorticoids. This inhibitory effect involves cross-talks between glucocorticoid and oxysterol signaling, and in particular between Pregnane X Receptor (PXR) and a common coactivator, PGC-1α. As a consequence of their inhibitory influence on sPLA2-IIA, glucocorticoids exacerbate the apoptotic effects of oxysterols on oligodendrocytes, resulting in secondary necrosis.

## Results

### The Glucocorticoid Receptor Inhibits the Stimulation of sPLA2-IIA by Oxysterols

**Figure 1:**
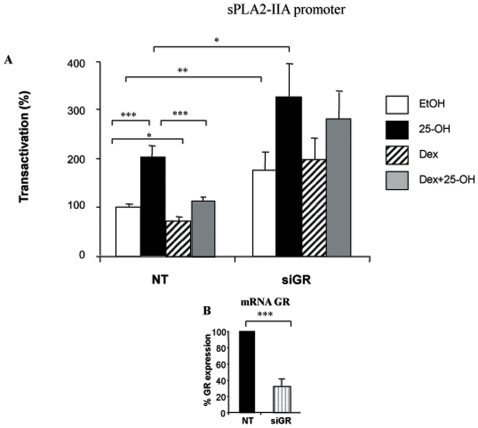
Effect of Dex and implication of the GR in sPLA2-IIA promoter activity. A/ 158N cells were transiently transfected with 0.2 µg of sPLA2-IIA(1 kb) and 0,1 µg of pRSV-βGal plasmids in the presence of 0.2 µg of mock vector (NT) or siRNA directed against the GR (siGR). Eighteen hours after transfection, cells were incubated with 25-OH (10^−5^ M) and/or Dex (10^−6^ M) for 24 h, and then luciferase and β-galactosidase activities were analyzed. Results are expressed as percentage of the basal activity; they represent the mean +/− SEM of 10 independent experiments performed in duplicate. In all experiments *p<0.05, **p<0.01, ***p<0.001 by using Bonferroni's test after ANOVA. B/ Test of the efficacy of the siRNA: Total RNA from 158N cells transfected with either non targeting siRNA or siRNA against GR was prepared. Real time RT-PCR was performed. 26S RNA was detected by specific primers and used to normalize GR expression levels.

We have previously shown that sPLA2-IIA is stimulated by oxysterols in an oligodendrocyte cell line. As glucocorticoids have anti-inflammatory actions, we examined whether they interfere with the regulation of sPLA2-IIA by oxysterols. First, we assayed the effects of Dex on sPLA2-IIA promoter activity. Oligodendrocytes were transiently transfected with a sPLA2-IIA promoter driving the expression luciferase reporter gene (sPLA2-IIA-Luc) and treated with 25-OH and/or Dex. While 25-OH (10 µM, 24 h) doubled the promoter activity (p<0.001), Dex treatment (1 µM, 24 h) inhibited by 30% the basal promoter activity (p<0.05) and blocked the stimulation by 25-OH (p<0.001) (figure 1A). The 25-OH amount that we have used in cell culture is comparable to that observed in the brain. As a matter of fact, we have previously shown that oxysterol levels are high in rat brain: 24S and 25-OH concentrations were about 25 µM and 0.16 µM, respectively [8].

Whether or not the GR was involved in this inhibition was assessed using a siRNA targeting the GR (siGR). Real-time PCR experiments showed that the siGR was able to knock-down GR expression (Fig. 1B). When we transfected the siRNA against the GR into these cells, the basal activity of the sPLA2-IIA promoter was enhanced as well as the stimulation by 25-OH, while the inhibitory effect of Dex was abolished. As expected, a mutated siRNA directed against the GR had no effect on the regulation of the sPLA2-IIA promoter (not shown). Conversely, overexpression of the GR inhibited the basal activity of the sPLA2-IIA promoter (not shown). These results show that the GR blocks the effect of oxysterols at the level of the PLA2-IIA promoter, suggesting an interference with oxysterol receptor signaling.

### Glucocorticoid Inhibition of PXR Expression

**Figure 2:**
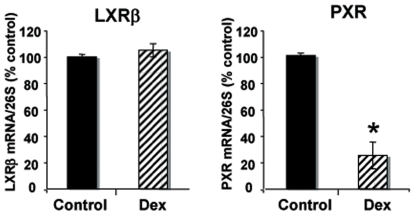
Effect of dexamethasone on the expression of LXRβ and PXR. 158N cells were incubated with Dex (1 µM) during 24 h. Total RNA was extracted and real-time PCR experiments were performed using primers recognizing specifically LXRβ or PXR. Results are expressed as the ratio of LXR or PXR expression over 26S. 100% is the level in control cells. They represent the mean +/− SEM of three independent experiments. *p<0.05 when compared to control by using Student's t test.

In order to understand the molecular mechanism of the cross-talk between GR and oxysterol receptors (LXR or PXR), we first investigated whether Dex can modify the expression of LXRβ and PXR receptors, previously shown to be involved in the regulation of sPLA2-IIA by oxysterols [8]. We therefore incubated the oligodendrocyte cell line 158N with Dex (1 µM) during 24 hours and analyzed by real time PCR the expression of LXRβ and PXR. As depicted in Figure 2, Dex did not alter the expression of LXRβ, but inhibited PXR transcript by 75%.

According to our previous results, PXR is only involved in the stimulatory effects of µM concentrations of oxysterols, thus, the selective reduction of PXR expression by Dex should only have an inhibitory influence on the stimulation of sPLA2-IIA by elevated levels of oxysterols. However, Dex also inhibits basal sPLA2-IIA promoter activity. This prompted us to search for additional mechanisms, such as competition for a common coactivator.

### Cofactor PGC-1α Is Involved in LXR and PXR Regulation of sPLA2-IIa Promoter

**Figure 3:**
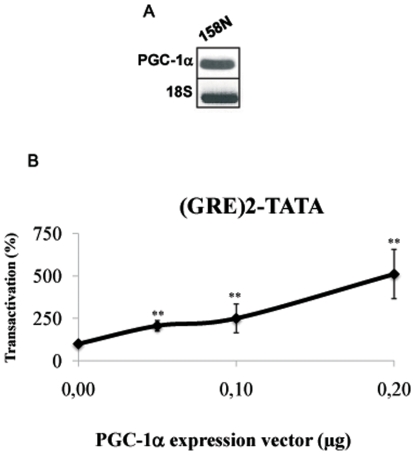
Implication of PGC-1α in GR transactivation. A/ Expression of PGC-1α. Total RNA was prepared from 158N cells. RT-PCR experiments were performed using primers recognizing specifically PGC-1α. PCR products were analyzed on agarose gel (2%) and visualized under UV. 18S RNA was detected by specific primers and used to normalize PGC-1α expression levels. B/ Implication of PGC-1α in the glucocorticoid pathway. 158N cells were transiently cotransfected with either 0.2 µg of (GRE)2-TATA-Luc, 0.1 µg of pRSV-βGal plasmids and increasing amounts of PGC-1α expression vector, as indicated. Eighteen hours after transfection, cells were incubated with Dex (10^−6^ M) for 24 h, and then luciferase and β-galactosidase activities were analyzed. Results are expressed as percentage of the basal activity and represent the mean +/− SEM of at least four independent experiments performed in duplicate. **p<0.01 when compared between cells transfected with PGC-1α using Bonferroni's test after ANOVA.

Another possible explanation for the inhibitory effect of the GR on sPLA2-IIA promoter activity is the competition with PXR (mediating the stimulation by micromolar concentrations of oxysterols) or LXRβ (involved in the regulation of basal promoter activity) for the recruitment of a common coactivator. We therefore intended to identify the coactivator implicated in the stimulation of sPLA2-IIA by 25-OH. To activate transcription, PXR and LXR need to recruit coactivators such as members of the p160 family (SRC-1, SRC-2/GRIP1/TIF2 and SRC-3/p/CIP/ACTR). Although the three p160 family members are expressed in oligodendrocytes, their overexpression or their knock-down by siRNA [24], [25] did not alter oxysterol stimulation of sPLA2 promoter (not shown). This observation ruled out the implication of the p160s in the stimulation of the sPLA2-IIA promoter by 25-OH in oligodendrocytes. We then tested whether PGC-1α, a common coactivator for LXR and PXR, could be involved in this pathway. RT-PCR showed that PGC-1α is indeed expressed in the oligodendrocyte cell line (Fig. 3A). First, we verified that PGC-1α is implicated in GR signaling in 158N cells. Overexpressing PGC-1α dose-dependently enhanced Dex-mediated transactivation of the glucocorticoid-sensitive promoter (GRE)2-TATA (5-fold enhancement of promoter activity when 0.2 µg of PGC-1α was transfected) (Fig. 3B).

**Figure 4:**
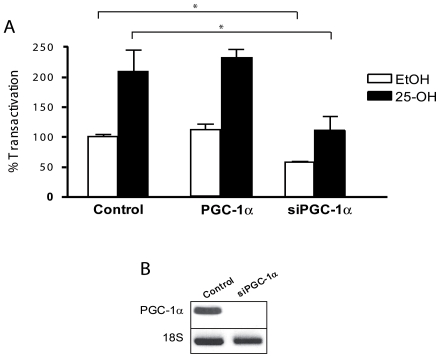
Implication of PGC-1α in sPLA2-IIA promoter activity. A/ Implication of PGC-1α in sPLA2-IIA promoter activity. 158N cells were transiently transfected with either 0.2 µg of sPLA2-IIA(1 kb), 0.1 µg of pRSV-βGal plasmids with PGC-1α expression vector, with mock vector or with a siRNA specifically directed against PGC-1α, as indicated. Eighteen hours after transfection, cells were incubated with 25-OH (10^−5^ M) for 24 h, and then luciferase and β-galactosidase activities were analyzed. Results are expressed as percentage of the basal activity and they represent the mean +/− SEM of at least four independent experiments performed in duplicate. *p<0.05 when compared between control cells and cells transfected with PGC-1α expression vector or siRNA against PGC-1α using Bonferroni's test after ANOVA. B/ The efficacy of the siRNA was analyzed by RT-PCR. 158N cells were transiently transfected with a siRNA specifically directed against PGC-1α. 24 hours after transfection, total RNA was prepared. RT-PCR experiments were performed using primers recognizing specifically PGC-1α. PCR products were analyzed on agarose gel (2%) and visualized under UV. 18S RNA was detected by specific primers and used to normalize PGC-1α expression levels.

We subsequently addressed the question of the implication of PGC-1α in the regulation of sPLA2-IIA promoter by 25-OH. Overexpression of PGC-1α did not further enhance the stimulatory effects of 25-OH (Fig. 4A). But interestingly, the knock-down of PGC-1α by siRNA significantly affected by 50% the basal activity (p<0.05) and the oxysterol-induced stimulation (p<0.05) of the sPLA2-IIA promoter (Fig. 4A). The efficacy of the siRNA directed PGC-1α was tested by RT-PCR (Fig. 4B) and WB (not shown).

**Figure 5:**
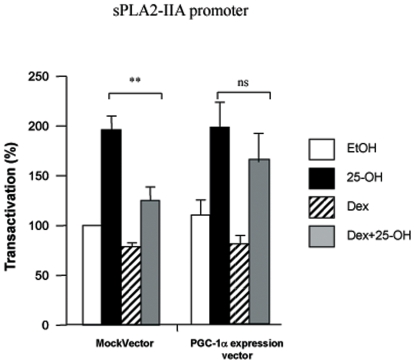
PGC-1α implication in sPLA2-IIA promoter regulation. 158N cells were transiently transfected with 0.2 µg of sPLA2-IIA(1 kb), 0.1 µg of pRSV-βGal plasmids and with 0.1 µg of PGC-1α expression vector or mock vector, as indicated. Eighteen hours after transfection, cells were incubated with 25-OH (10^−5^ M) and/or Dex (10^−6^ M) for 24 h, and then luciferase and β-galactosidase activities were analyzed. Results are expressed as percentage of the basal activity; they represent the mean +/− SEM of at least four independent experiments performed in duplicate. **p<0.01, when comparing between 25-OH and Dex+25-OH using Bonferroni's test after ANOVA.

The competition for a common coactivator, PGC-1α, which is present at limiting amounts in the cells, could be part of the cross-talk between GR and LXR/PXR. To test this hypothesis, we transfected 158N cells with the sPLA2-IIA promoter and overexpressed PGC-1α. Then, the cells were treated with 25-OH in the presence or absence of Dex. As expected, Dex repressed the effects of 25-OH at the level of the sPLA2-IIA promoter. Interestingly, when we enhanced the intracellular amounts of PGC-1α, Dex was not able to repress 25-OH effects any more (Fig. 5).

### Cross-Talk between 25-OH Oxysterols and Dexamethasone on Apoptosis

We have formerly described that sPLA2-IIA partially protects oligodendrocytes from 25-OH-triggered apoptosis. As a matter of fact, incubation of the oligodendrocyte 158N cell line with a conditioned medium enriched with sPLA2-IIA to some extent protected from the deleterious effects of 25-OH. Since Dex inhibits sPLA2-IIA, it could be expected to exacerbate the apoptotic effects of oxysterol. We thus studied the combined effects of Dex and 25-OH on oligodendrocyte apoptosis.

**Figure 6:**
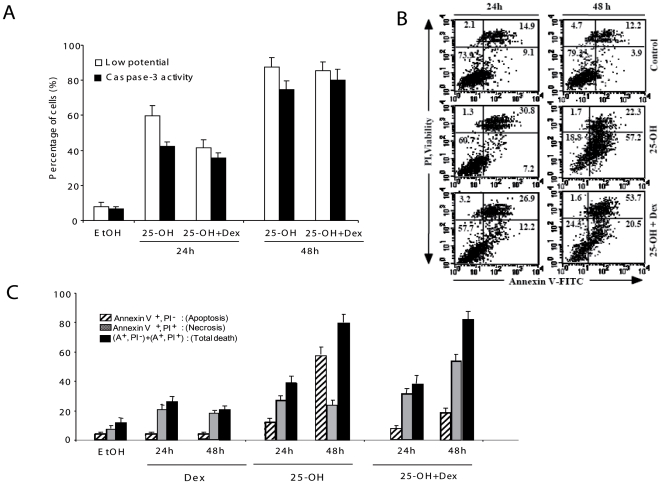
Cross-talk between oxysterols and glucocorticoids in oligodendrocyte death. A/ Histograms showing cells treated with or without 25-OH in presence or absence of dexamethasone. As described here, the mitochondrial membrane potential was determined by DiOC6(3) and propidium iodide staining of the cells, followed by flow cytometry analysis. The different categories of cell populations as described in (A) have been simultaneously plotted. In all cases, 10.000 cells have been counted. The standard deviations have been extracted from five different experiments. B/ Biparametric flow cytometric analysis of oligodendrocytes treated with or without 25-OH in presence or absence of dexamethasone. Apoptotic cells were identified as Annexin V-FITC positive/PI negatives, whereas necrotic cells or cells exhibiting secondary necrosis were considered as Annexin V-FITC positive and PI positive. The addition of the two populations gives the total cell death in the present conditions. A typical experiment with cells treated for 24 h or 48 h is presented. 10.000 cells have been counted for each representation (the cell debris and aggregated cells have been excluded from the analysis by selection based on their light scattering properties). C/ Histograms of the cells treated with or without 25-OH in the presence or absence of dexamethasone. The different categories of cell populations as described in (B) have been simultaneously plotted. In all cases 10.000 cells have been counted. The standard deviations have been extracted from five independent experiments.

Oligodendrocytes were incubated with 25-OH during 24 and 48 h with or without co-treatment with Dex. Afterwards, we analyzed by flow cytometry several phenomena occurring during apoptosis: mitochondrial membrane potential (early apoptotic event), caspase 3 activity (late event), Annexin V staining (in order to detect phosphatidyl serine exposure in apoptotic cells) and propidium iodide staining (a DNA intercalating agent). As expected, the number of cells with low mitochondrial membrane potential dramatically increased after 24 h and 48 h of 25-OH treatment (59.7% at 24 h, 87.9% at 48 h *vs* 7.8% in the control conditions) (Fig. 6A). The drop of mitochondrial membrane potential appears to be associated with caspase-3 activation (Fig. 6A). Annexin V and propidium iodide staining, which respectively indicate apoptosis (% of cells with A^+^PI^−^) or necrosis (% of cells with A^+^PI^+^), correlated with the previous observation: 3.8% of 158N cells were in apoptosis in control conditions, while 14.2% and 57.7% underwent apoptosis after 24 h and 48 h of 25-OH treatment, respectively (Fig. 6B). Interestingly, a time-dependent effect of 25-OH was not observed on the number of cells undergoing necrosis (A^+^/PI^+^ cells).

Dexamethasone alone did not affect mitochondrial membrane potential, caspase 3 activity (not shown), but slightly enhanced the number of necrotic cells (Fig. 6C). A combined treatment with Dex and 25-OH was able to partially rescue from the increase in the number of cells with low mitochondrial membrane potential after 24 h of treatment (59.7% in 25-OH *vs* 40% in 25-OH + Dex conditions) but did not modify the caspase-3 activation (Fig. 6A). After 48 h, Dex+25-OH cotreatment did not reverse the increase in the mitochondrial membrane potential nor caspase-3 activity (Fig. 6A).

The time-dependent apoptotic effect of 25-OH was hammered by Dex treatment (Fig. 6B–6C). Nevertheless, the total number of dead cells was not affected by the combination of Dex+25-OH (*vs* 25-OH alone). To understand this discrepancy, we have analyzed by flow cytometry the percentage of necrotic cells (Percentage of PI^+^/A^+^ cells; Fig. 6B). We noticed that treatment of 158N cells with 25-OH and Dex elicited an increase in cell necrosis, which probably corresponds to a so-called “secondary necrosis”.

### Alteration of Oligodendrocyte Morphology and Cytoplasm by 25-Hydroxycholesterol and Dexamethasone

**Figure 7:**
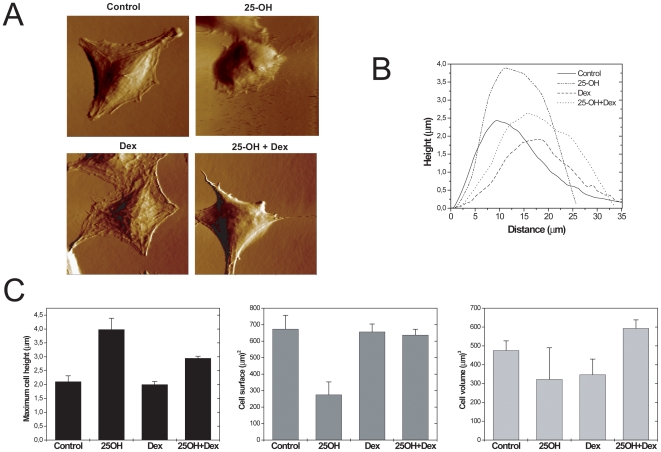
Alteration of oligodendrocytes morphology by 25-OH and Dex. A/ Representative AFM deflection images of living oligodendrocytes in culture medium. 158N cells were cultured on a matrix of collagen I during 24 h and then cells were incubated or not with 25OH (10^−5^ M) and/or Dex (10^−6^ M) during 24 h. The cells were then imaged with AFM in contact mode. This experiment was repeated three times, and typical images are presented here. Image scan size: 60 µm ×60 µm. B/ Height profiles corresponding to the cross-sections of the same cells as in (A). C/ Histogram of the morphometric parameters, *i.e.* maximum cell height, cell surface and total cell volume of 158N cells, incubated or not. All averages are calculated from five cells.

Our previous data [8] showed that 25-OH alters oligodendrocytes morphology. The effects of 25-OH and Dex on the shape of oligodendrocytes were monitored by atomic force microscopy (AFM), a technique that provides topographic images as well as morphological parameters (height, surface, volume) of individual attached living cells [26], [27]. Figure 7 shows typical deflection images of living 158N cells, obtained in contact mode, cultured in the absence or presence of 25-OH (10^−5^ M) and/or Dex (10^−6^ M) during 24 h. Cytoskeleton elements are clearly visible underneath the plasma membrane of control cells. The exposure of 158N cells to 10 µM 25-OH during 24 h resulted in striking alterations of cell morphology and important variations both in height and width (figure 7B). As depicted in Figure 7C, treatment with 25-OH elicited a significant increase of the cell maximum height from 2.11±0.21 µm to 3.98±0.40 µm, an edge retracted by approximately 10 µm and a spherical shape enhancement. The surface of the 25-OH-treated cells was reduced by 59% compared to the control, whereas the total volume was diminished by 32%.

There was no significant effect of Dex alone on the morphology or on the cell surface and the shape of oligodendrocyte cell line. These morphometric alterations elicited by 25-OH were reversed by co-treatment with Dex (Figure 7A). Figure 7C shows that with a combined treatment with 25-OH and Dex, the cell surface and volume were brought back to comparable values as control condition. Dex+25-OH treatment provoked a decrease in oligodendrocyte height comparing to 25-OH condition. Nevertheless, after incubation with Dex+25-OH, the ratio cell volume/surface increased as in the case of treatment with 25-OH, indicating a slight swelling of the cell. Besides, cytoskeleton elements were not visible any more underneath the plasma membrane of the cells. These morphometric data show that 25-OH and Dex provoke profound, but different, modifications in oligodendrocyte cell line shape. We tried to image by AFM the oligodendrocytes after 48 h of incubation with 25-OH and/or Dex. However, we were not able to have accurate images of the cells at this time point because the cells are in a very bad condition due to the increase of the number of dead cells or cells undergoing apoptosis/necrosis. At 48 h, the cells detached rapidly when the AFM tip touches the ‘living’ remaining cells. As we have precoated the dishes for AFM experiments, we have verified that this coating did not alter neither the apoptotic/necrotic signal nor the sPLA2-IIA regulation by Dex and/or 25-OH (not shown).

**Figure 8:**
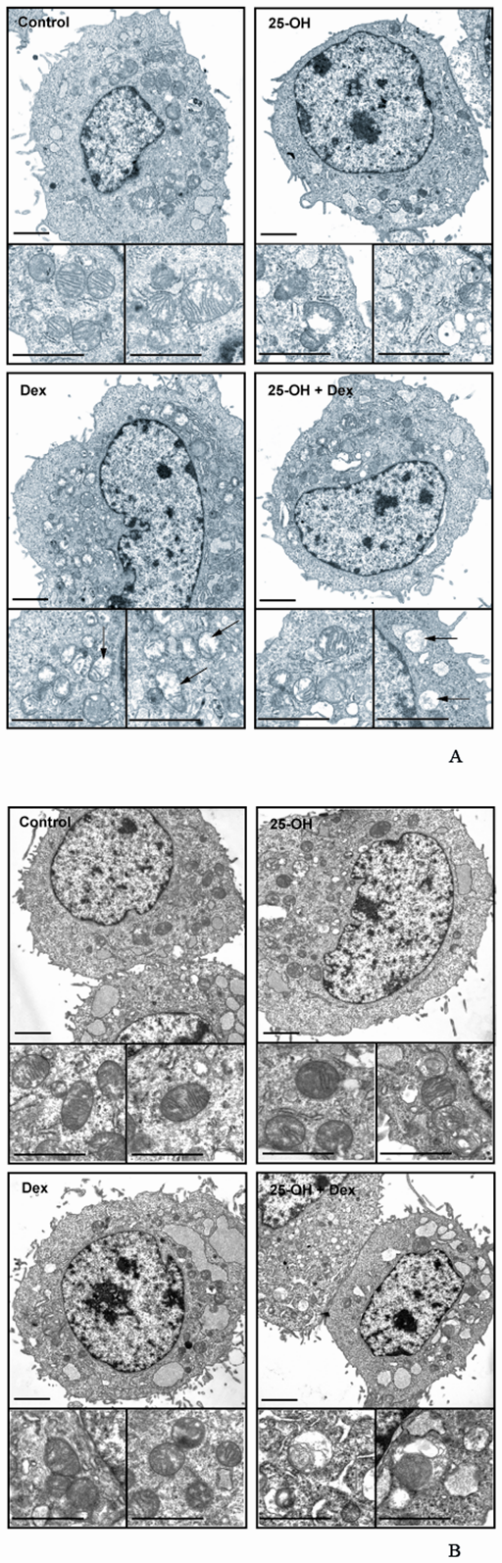
Alteration of oligodendrocytes cytoplasm by 25-OH and Dex. Transmission electron micrographs of 158N cells incubated or not with 25OH (10^−5^ M) and/or Dex (10^−6^ M) during 24 h (A) or 48 h (B). The cells were fixed with 2.5% glutaraldehyde in 0.1 M cacodylate buffer (pH 7.2) for 1 h at 4°C and TEM was performed. Insets: magnification showing representative mitochondria. By comparison with controls, mitochondria cristae seem to disappear. They partly lost their normal structure (arrows). Scale bar = 2 µm.

To further analyze the effects of 25-OH and Dex on oligodendrocytes, we performed electron microscopy of oligodendrocytes incubated with 25-OH and/or Dex. In control conditions, mitochondria have a typical conformation with normal cristae membranes within an unaffected cytoplasm (Figure 8A). The addition of 25-OH modifies the cytoplasm, which becomes clearer with a slight swelling of both the Golgi apparatus and the endoplasmic reticulum (ER). The mitochondria are also affected: they become electron dense and exhibit some intramitochondrial lumen. The treatment with Dex seems to affect firstly the mitochondria that appear translucide and lose their typical conformation (cristae membrane disappearance). The combined treatment with 25-OH and Dex induces a dramatic swelling of the Golgi apparatus, associated to a minor extent with ER swelling. The cytoplasm is also disorganized. Curiously, some mitochondria seem to preserve their conformations whereas others look as if they have been subjected to microautophagy (elimination of unwanted ROS-producing mitochondria).

We have performed TEM after 48 h of treatment with Dex and/or 25-OH (Fig. 8B). Dex (48 h) treated cells have similar, but more pronounced, stigmata than at 24 h: the mitochondria are affected and the ER is dilated. With 25-OH there an important presence of autophagosomes and the mitochondria are affected. The treatment with 25-OH + Dex leads to the clear occurrence of an important mitophagy, taking place in a totally disordered cytoplasm with widely enlarged ER and lumen at the level of the nuclear envelop.

## Discussion

Oxysterols are important molecules for brain homeostasis, and their levels are altered in several neurodegenerative disease like AD and demyelinating diseases (MS, Smith-Lemli-Optiz disease) [5], [6], [28]. Our previous data showed that oxysterols, and in particular 25-OH, can provoke oligodendrocyte apoptosis, but also stimulate the synthesis of the sPLA2-IIA with partial protective effects [8]. In this study, we have analyzed the effects of the glucocorticoid Dex (often used as anti-inflammatory agent) on oxysterol-induced apoptosis and sPLA2-IIA induction. We have demonstrated that Dex blocks sPLA2-IIA stimulation by oxysterols, an effect which is mediated by the GR. As a matter of fact, GR silencing causes an increase in sPLA2-IIA basal activity as well as its induction by oxysterols. Moreover, it abolishes Dex inhibitory effects towards sPLA2-IIA. Dex negative effects on 25-OH are restricted to sPLA2-IIA because they did not affect the expression of the cholesterol transporter (ABCA1), another 25-OH target gene (not shown).

We have demonstrated two mechanisms of action that could account for the cross-talk between GR and oxysterol receptors in oligodendrocytes: (i) alteration by the GR of LXRβ or PXR expression; (ii) competition between LXR and GR for a common coactivator. We have previously shown that oligodendrocytes are able to produce oxysterols that act by an autocrine/paracrine loop via LXRβ and increase the basal activity of the sPLA2-IIA promoter, while PXR is responsible for the stimulation of sPLA2-IIA by higher amounts of added 24(S)-OH or 25-OH. PCR experiments demonstrated that PXR expression, but not LXRβ, expression was affected by Dex, which is in accordance with findings of Greger *et al* in liver [29]. We also explored the second possibility, namely competition for a common coactivator between the GR and LXR-PXR. As p160s are not implicated in the regulation of sPLA2-IIA promoter by 25OH, we focused our attention on PGC-1α, another common coactivator. We have shown that PGC-1α knock-down altered 25OH signaling at the level of the sPLA2-IIA promoter. Furthermore, PGC-1α overexpression enhanced glucocorticoid signaling at the level of a classical (GRE)2-TATA construct. Consequently, PGC-1α is implicated in both signaling pathways in 158N cells. We overexpressed PGC-1α in the oligodendrocyte cell line, and we obtained a reduction in the repressive effect of Dex at the level of sPLA2-IIA promoter. These results suggest that GR competes with LXR/PXR for a common limiting coactivator, PGC-1α, a versatile coactivator that interacts with numerous nuclear receptors, including LXR, PXR and GR. This kind of competitive mechanism has already been described between PXR and HNF-4, which struggle for PGC-1α [30]. As Dex is also well known to be a common ligand for PXR and GR, one could wonder if the cross-talk between these receptors is due to the ability of Dex to bind to both of them. Dex and 25-OH effects are opposite (25-OH activates sPLA2-IIA promoter while Dex represses it). We have shown by using siRNA against either GR or PXR that the stimulation of sPLA2-IIA by 25-OH is PXR-dependent, while the repression by Dex is GR dependent. A siRNA directed against PXR did not relief from Dex inhibition (not shown) but a siRNA targeting GR blocked Dex inhibitory effects. Thus, the cross-talk between PXR and GR is not mediated by the binding of Dex to both receptors.

There is increasing evidence in favor of the existence of a cross-talk between oxysterol and glucocorticoid signaling pathways. LXRs can strongly down-regulate the expression and activity of 11β-hydroxysteroid deshydrogenase type I (11β-HSD-1) [31], which controls the concentration of active glucocorticoids by converting inactive corticosteroids into biologically active ones. Moreover, 24(S)-OH was shown to block the enzymatic activity of 11β-HSD. LXRs are also implicated in the control of steroid metabolism. Hypercorticosteronemia has been described in LXR deficient mice [32], and these mice overexpress several genes implicated in steroid metabolism (StAR, CYP11A1 and 3β-HSD-1). Furthermore, LXR agonist reduces GR expression [33], whereas GR in turn can suppress LXR expression [34]. The present study adds two novel mechanisms to the cross-talk between glucocorticoids and oxysterols, namely, the inhibition of PXR expression and the competition for PGC-1α.

What is the physiological impact of this cross-talk? We have shown that 25-OH provokes oligodendrocyte apoptosis by an LXR independent manner. At the same time, 25-OH activates sPLA2-IIA by means of the LXR/PXR, with a partial protective effect towards oligodendrocytes. Thus, we put forward that activation of sPLA2-IIA may be able to counterbalance the apoptotic effects of 25-OH. Since Dex can block the activation of sPLA2-IIA by 25-OH, it can be expected to dampen oligodendrocyte defenses and to accelerate cell death. This hypothesis is supported by the enhancement of the number of necrotic cells after 48 h of treatment with Dex+25-OH, which is in favor of a secondary necrosis that occurs after an acceleration of the apoptotic process. A second line of evidence confirms this observation. We have assayed the production of superoxide anions, which are reactive oxygen species (ROS). While in control cells the percentage of superoxide anion is about 15%+/−3%, 25-OH incubation augmented the amount of superoxide anion (29%+/− 3% at 24 h and 23%+/−4% at 48 h). Interestingly, when oligodendrocytes were incubated with Dex and 25-OH, we detected a larger amount of cells producing superoxide anion (35%+/−5% at 24 h and 57%+/−7% at 48 h). The death of oligodendrocyte under Dex+25-OH conditions takes place in a context where the production of ROS is increased. This observation could explain the shift from apoptotic (25-OH) to necrotic (25-OH+Dex) death.

Furthermore, arguments obtained from atomic force microscopy as well as electron microscopy sustain this assumption. We have demonstrated that 25-OH and Dex profoundly modify oligodendrocyte shape, cytoplasm and mitochondria. After incubation with 25OH, the shape of the oligodendrocytes became spherical, suggesting the loss of focal adhesion sites that prepares the detachment of the cells. This was accompanied by mitochondrial defects and a slight swelling of both the Golgi apparatus and the endoplasmic reticulum. The treatment with Dex did not modify cell shape but affected the mitochondria. The combined treatment with 25OH and Dex gave the impression of an improvement of cell external morphology. Nevertheless, oligodendrocyte shape is altered and their cytoplasm is also profoundly disorganized. These morphological observations corroborate a switch to oligodendrocyte necrosis.

In conclusion, we have shown that Dex is able to accelerate the death of oligodendrocytes by inhibiting sPLA2-IIA. Apoptosis was initiated by 25-OH and sped up and shifted to secondary necrosis by glucocorticoid in order to get rid of the non-functional oligodendrocytes. These phenomena are orchestrated by nuclear receptors (LXR, PXR and GR) that compete with a common coactivator PGC1α on the level of sPLA2-IIA promoter.

## Materials and Methods

### Cell Culture

The immortalized mouse oligodendrocyte cell line 158N, which has preserved oligodendrocyte characteristics and expresses myelin proteins [35], was maintained in Dulbecco's minimal essential medium (DMEM) supplemented with 5% fetal calf serum (Gibco), 100 U/mL penicillin and 100 mL/mL streptomycin (Gibco).

### Plasmids

The Luciferase construct containing the sPLA2-IIA promoter has already been described [36]. The (GRE)2-TATA-luc plasmid was described by Grenier *et al*. [24]. The siRNAs directed against PGC-1α and GR as well as control siRNA (non targeting (NT) siRNA) were purchased from Dharmacon (Lafayette, CO). PGC-1α was graciously provided by Dr JF Louet (Houston, TX, USA).

### Transient Transfections

158N cells were transiently transfected using Effecten reagent (QIAGEN). One day before the transfection, 158N cells (1.5×10^5^ cells/well) were seeded into 6-well plates and incubated in DMEM culture medium containing 5% fetal calf serum. The sPLA2-IIA-luc plasmid (0.3 µg), the pRSV-βGalactosidase expression vector (0.1 µg) and the coactivator expression vectors, mock vectors or siRNAs at the concentrations indicated in the figure legends were mixed with a solution containing Effecten reagents (0.85 mg/ml) in serum-free DMEM, as suggested by the manufacturer. The mixture was then added to the cells overnight. One day after the transfection, the medium was replaced by DMEM containing 5% charcoal-treated fetal calf serum containing or not 25-hydroxycholesterol or 24(S)-hydroxycholesterol (10 µM) and/or the GR agonist dexamethasone (1 µM) during 24 hours.

Luciferase activity was determined using the enzymatic method described in Massaad *et al*. [37]. The β-galactosidase activity was used to normalize the transfection efficiency and measured as described in Tallec *et al* [38].

### Quantitative and Non Quantitative RT-PCR

Total RNA from cultured 158N cells was obtained using RNA now (Ozyme, France) and 1 µg was reverse transcribed with random primers from Biolabs (Beverly, MA) and Reverse Transcriptase M-MuLV-RT from Finnzymes (Espoo, Finland). PCR experiments were performed using Taq DNA polymerase purchased from Biolabs (Beverly, MA) and primers specific for each gene from Proligo (Boulder, CO). PCR products were analyzed on agarose gel (2%) and visualized under UV.

Quantitative PCR was performed with standard protocols using SYBR®Green (ABgene, France) as fluorescent detection dye in ABI PRISM® 7000 in a final volume of 25 µl which also contained 300 nM primers (Eurofins Genomics Operon, Orsay, France) and 20 ng of reverse transcribed RNA in 96-well plates. For the characterization of the generated amplicons and to control the contamination by unspecific by-products, a melting curve analysis was applied. Each reaction was performed in triplicate and the mean of at least three independent experiments was calculated. All results were normalized to the 26S mRNA level and calculated using the Delta Ct method. The primer sequences used in real time PCR are listed below:

PGC-1α sense: CCTTGCCATTGTTAAGACC;

PGC-1α antisense: TGCTGCTGTTCCTGTTTTT;

GR sense: CAAAGGCGATACCAGGATTC;

GR antisense: TCAGGAGCAAAGCATAGCAG;

LXRβ sense: CTTGGTGGTGTCTTCTTGA;

LXRβ antisense: TGTGGTAGGCTGAGGTGTA;

PXR sense: CAAGGCCAATGGCTACCA;

PXR antisense: CGGGTGATCTCGCAGGTT;

26S sens: AGGAGAAACAACGGTCGTGCCAAAA;

26S antisens: GCGCAAGCAGGTCTGAATCGTG.

18S sense: CTCGGGCCTGCTTTGAACAC;

18S antisense:CTACCACATCCAAGGAAGGC

### Study of Apoptosis by Flow Cytometry

Changes in mitochondrial membrane potential difference (ΔΨm) were evaluated by incubating cells (Oligodendrocytes, 5×10^5^/ml) for 15 min at 37°C, with DiOC_6_(3) (stock solution 1 µM in ethanol, final concentration 10 nM). Cells were then analysed by using the FACS calibur cytometer. The fluorescence was collected after suitable compensation, in FL-1 (530±30 nm) channel for DiOC6(3) together with the PI permeability assay recorded in FL3 (PL; long pass>670 nm).

Late apoptotic events and/or secondary necrosis were estimated through the estimation of the exposed phosphatidylserine (PS) on the outer plasma membrane leaflet by staining cells with annexin V-FITC (1 µg/ml) for 10 min at 4°C. The annexin V-FITC absorption on cell surface was monitored by flow cytometry in FL-1 channel (530±30 nm), together with the membrane integrity assay using propidium iodide (PI, 1 µg/ml), which emitted in FL-3 channel (long pass>670 nm), as classically described.

Caspase-3-like activity was assayed by flow cytometry in intact cells incubated with the cell-permeable fluorogenic caspase substrate PhiPhilux G1D2 (OncoImmunin Inc., Kensington, MD, USA), which contains the GDEVDG sequence. Briefly, oligodendrocytes cells were harvested and washed twice in PBS buffer. Cells were resuspended in 50 µl of PhiPhilux-containing solution and incubated for 1 h at 37°C in the dark. Then, they were washed and suspended in PBS buffer. Fluorescence was detected in FL-1 channel together with the membrane integrity assay using PI (1 µg/ml) as described above.

Superoxide anion production was assayed on treated cells incubated with 2.5 µM dihydroethidium for 15 min at 37°C for, and then washed and resuspended in phosphate-buffered saline pH 7.2. They were counterstained with 2 µM TOPRO-3 (1 mg/ml stock solution) to monitor cell viability just before flow cytometry analysis. The fluorescence was collected after excitation at 488 nm in the FL-2 channel (585±42 nm) for HE and after exciation at 635 nm in the FL-4 channel (661±16 nm) channel for TOPRO-3.

### Atomic Force Microscopy

AFM measurements were performed on a commercial AFM Bioscope (Nanoscope IIIa, Digital Instruments, Veeco, USA) in contact mode. Using a fluid cell, AFM contact mode images of living cells were recorded in culture medium using a long V-shape silicon nitride cantilever (320 µm in length), with a nominal spring constant of 0.01 mN/m (MLCT-AU microlevers, Veeco, France) to minimize the force applied to the cells. The image fields (60 µm×60 µm) were obtained at 0.5 Hz; 6 to 10 min were required to scan the entire sample. 158N cells cultured on collagen I (rat tail, BD Biosciences, France) coated Petri dishes were mounted directly on the stage of the inverted microscope (Olympus IX 70, France) of the Bioscope to choose viable cells at relative low density. The samples were equilibrated in the cell medium for a 10 to 15-minute period to avoid problem with drift of the equipment.

Morphometric parameters (maximum height, cell surface and cell volume) were determined from the AFM topography images using the Nanoscope software (v5.12). First, the individual image was plane-fitted and then the total surface and volume of the cell were analyzed using the bearing software feature. Individual cell volume was based on the following principle: each pixel included information on three dimensions. The x and y parameters are given by the size of the scan area (50 µm or 60 µm in our case) and by the number of scan lines per image (512 here). The z (height) information is given by the specific color level of each pixel. Thus, after selection of the “base area” of the cell by a threshold procedure, the total volume of the cell was simply obtained by integrating the height over the base area.

### Transmission Electron Microscopy

Cells were fixed with 2.5% glutaraldehyde in 0.1 M cacodylate buffer (pH 7.2) for 1 h at 4°C, followed by 1% OsO4 for 1 h at 4°C. Subsequently, cells were contrasted in 1% Uranyl acetate, dehydrated through a graded series of acetonitrile and embedded in epoxy resin. Thin sections (100 nm) were cut on a Reichert Ultracut E ultramicrotome, placed on 100-mesh copper grids, contrasted in uranyl acetate and lead citrate and viewed under a Hitachi H600 electron microscope (Tokyo, Japan).

### Statistical Analysis

Unless otherwise specified, means of treatment groups were compared by one-way analysis of variance (ANOVA). When the ANOVA showed that there were significant differences between the groups, Bonferroni's test was used for between-group comparisons. Student's t test was used for the statistical comparison between two groups. A p value ≤0.05 was considered to be statistically significant.
